# Cross-Union Surgery for Congenital Pseudarthrosis of the Tibia

**DOI:** 10.3390/children8070547

**Published:** 2021-06-24

**Authors:** Claire E. Shannon, Aaron J. Huser, Dror Paley

**Affiliations:** Paley Orthopedic and Spine Institute, West Palm Beach, FL 33407, USA; ahuser@paleyinstitute.org (A.J.H.); dpaley@paleyinstitute.org (D.P.)

**Keywords:** congenital pseudarthrosis of the tibia (CPT), congenital pseudarthrosis of the fibula, neurofibromatosis 1, NF1, cross-union, anterolateral bowing, hamartoma, bisphosphonate, zoledronic acid, bone graft, BMP

## Abstract

Congenital Pseudoarthrosis of the Tibia (CPT) is a rare condition with a reputation for recurrent fractures and failure to achieve union. A large variety of surgical procedures have been attempted for the treatment of fractured cases of CPT with an average rate of union without refracture of only 50%. Intentional cross-union between the tibia and fibula has been reported to improve these results to 100% union with no refractures. This is a retrospective study of 39 cases of CPT in 36 patients treated by the Paley cross-union protocol with internal fixation, bone grafting, zoledronic acid infusion and bone morphogenic protein 2 (BMP2) insertion. All 39 cases of CPT united at the tibia and developed a cross-union to the fibula. Two patients had a persistent fibular pseudarthrosis, one that was later treated at the time of planned rod exchange and one that has remained asymptomatic. There were few postoperative complications. There were no refractures during the up to 7-year follow-up period. The most common problem was the Fassier-Duval (FD) rod pulling through the proximal or distal physis into the metaphysis (66.7%). This did not negatively affect the results and was remedied at the time of the planned rod exchange. The Paley Cross-Union Protocol is very technically demanding, but the results have radically changed the prognosis of this once sinister disease.

## 1. Introduction

Congenital Pseudoarthrosis of the Tibia (CPT) is a rare condition with an incidence between 1:140,000 to 1:250,000 live births [1]. A diagnosis is often made early in life due to an anterolateral bowing deformity of the affected leg or a pathologic fracture of the tibia and/or fibula. CPT is most commonly associated with neurofibromatosis (NF) in 50–90% of cases [2]. Associations with fibrous dysplasia and osteofibrous dysplasia have been reported [3,4]. Non-operative management with protective bracing is considered the standard of care in cases that have not yet broken. Once the bone has fractured, surgical management is indicated to achieve union and prevent refracture. A wide variety of surgical treatments have been tried for the management of CPT, with variable success. As a result of these factors, there has been a general acceptance that refracture or failure to achieve union is a common pathway in this condition’s natural history, with failure rates (lack of union or union followed by refracture) as high as 68% [5,6]. The average rate of union without refracture for all of these methods is approximately 50% [2,7]. Persistent nonunion or refracture leads to additional surgery and secondary changes in the leg, including ankle and knee contractures, malalignment, leg length discrepancy, calcaneo-cavus foot deformity, foot length shortening, calf muscular atrophy, gait alterations, coxa valga, and even hip dysplasia [8]. Beyond the physical and mental trauma that results from the interruption of childhood with repeated surgery, the likelihood of failed treatment may lead to the recommendation for amputation as a primary or secondary treatment [9].

**Figure 1:**
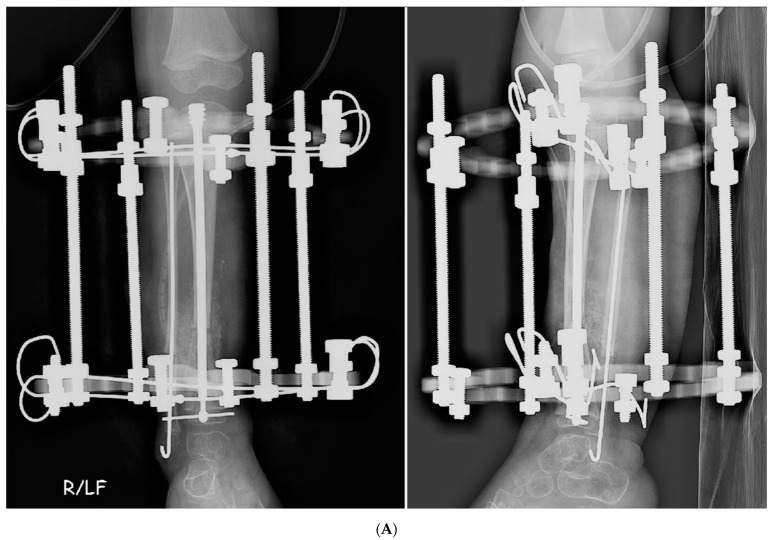

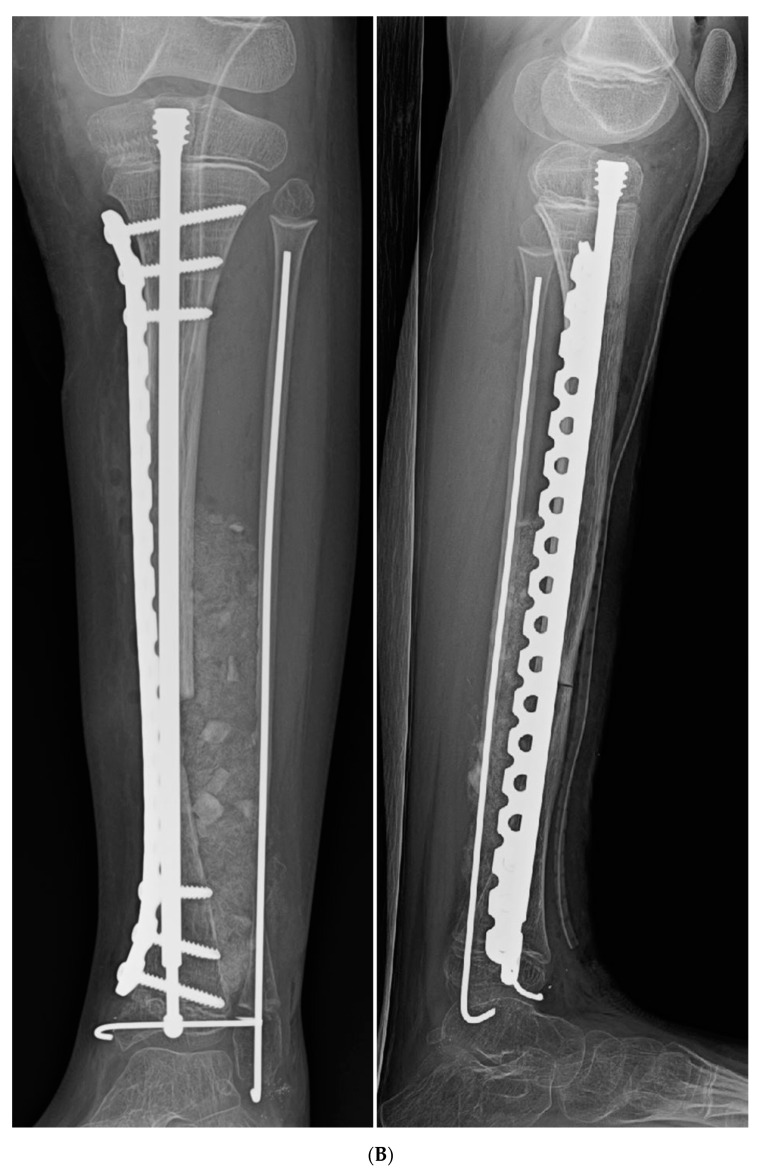
(**A**) AP and lateral radiographs that were taken postoperatively of a patient who underwent a Paley cross-union procedure performed with a circular external fixator. (**B**) AP and lateral radiographs that were taken postoperatively of a patient who underwent a Paley cross-union procedure performed with all internal fixation.

In 2011, Choi et al. [10] and in 2012, Paley [11] independently published their respective methods of treating patients with CPT by creating an intentional cross-union between the tibia and fibula. Choi et al. did this by converging the fibula towards the tibia, bone grafting between them and fixing the tibia by IM rodding across the ankle joint and circular external fixation (4-in-1 technique). Paley achieved this by rodding both bones straight without crossing the ankle and bone grafting between the tibia and fibula to create a wide bridge of bone, applying a circular external fixator, using bone morphogenic protein 2 (BMP2) intraoperatively and using Zoledronic Acid preoperatively and 3 months postoperatively (Paley cross-union protocol) (Figure 1A). Both achieved union in all cases without refracture. This 100% success rate of union without refracture was double that of all previous methods. Furthermore, the age at the time of treatment was not a limitation to success. In 2019, Paley published a modification of his original method substituting a malleable plate on the tibia for the circular external fixator [8] (Figure 1B). The Paley cross-union protocol was published with step-by-step illustrations in the supplement to this article [12]. The purpose of this study is to examine the early results of the Paley cross-union protocol using only internal fixation (no external fixation).

## 2. Materials and Methods

Institutional review board approval was obtained for a retrospective review of charts and radiographs from February 2014 to April 2019. Patients were included if they had a diagnosis of CPT, underwent the Paley cross-union protocol with all internal fixation, and had chart/radiographic documentation to support a minimum of 24-month follow-up. There was a total of 39 cases of CPT in 36 patients treated by the Paley cross-union internal fixation that met the inclusion criteria. No patients in the treatment group were excluded from the study. There were 16 boys and 20 girls. The mean age at the time of surgery was 3.3 years (range 12 months–13.5 years). The mean follow-up was 35 months (range of 24–85 months). Sixty-four percent of patients had a clinical diagnosis of NF1, 6% had a diagnosis of osteofibrous dysplasia, and 30% were idiopathic or without a diagnosis. Seven of the 36 patients (19%) had undergone previous failed surgery for CPT.

**Figure 2:**
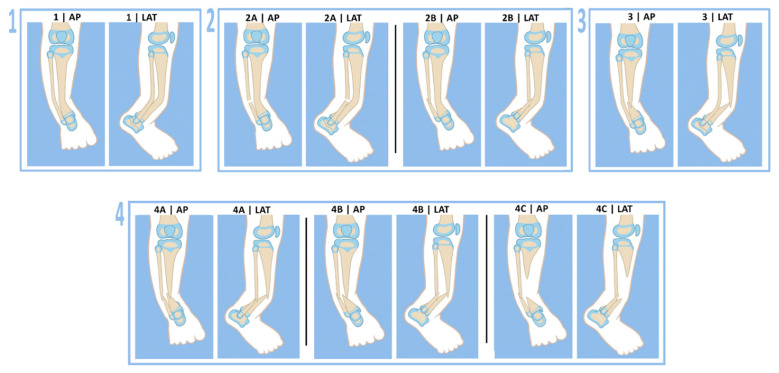
The Paley classification of CPT: (**1**) intact tibia and fibula with anterolateral bow; (**2A**) intact tibia with broken fibula remaining at station; (**2B**) intact tibia with broken fibula that has proximally migrated; (**3**) Intact fibula with broken tibia; (**4A**) Broken tibia and fibula with fibula remaining at station; (**4B**) broken tibia and fibula with proximal migration of fibula; (**4C**) broken tibia and fibula with significant bone defect.

All cases of CPT were classified radiographically using the Paley classification (Figure 2). There were five type 1, one type 2a, six type 2b, eight type 3, six type 4a, twelve type 4b, and one type 4c. All patients were given an infusion of Zoledronic acid infusion prior to cross-union surgery. All but one patient was given a second infusion approximately 12 weeks post-cross-union surgery. The one exception developed transient pancytopenia after the first infusion making a second infusion contra-indicated. Fortunately, this patient fully recovered spontaneously without any further complications. BMP2 was used in all but one patient who had a prior history of cerebral glioma. Since BMP2 is not FDA-approved in children, it was used in an off-label fashion. All parents/guardians were informed of this off-label use of BMP2. Informed consent, including the theoretical risk of oncogenesis secondary to BMP2, especially in NF1 patients, was obtained from all parents/guardians.

The surgical technique was performed as previously described by the senior author (D.P.) [12,13]. A Fassier-Duval nail (Pega Medical, Montreal, QC, Cananda) was used in all but one case, where a SLIM rod (Pega Medical, Montreal, QC, Cananda) was used at the index procedure since the child was nearing skeletal maturity. A malleable EVOS plate (Smith & Nephew, Memphis, TN, USA) was used in all but the first case (Smith & Nephew VLP plate used as EVOS had not been released yet). The fibula was rodded with a wire (1.1, 1.5, or 1.8 mm) or SLIM Rod in all cases. The senior author performed or assisted in all of the cross-union surgeries.

Three radiographic series were analyzed for each patient: preoperative, immediate postoperative and most recent follow-up. These included anterior-posterior (AP) and lateral views of the tibia and a long AP of both lower extremities from hips to ankles (either a lying pull-down view or a standing view) preoperatively, and if available, at most recent follow-up (19 patients). The radiographic analysis included the healing status of the tibia and fibula, which was defined as united if the osteotomy/pseudarthrosis lines were no longer present and/or a bridging cross-union above and below the CPT site was present. Frontal plane deformity parameters, including limb length discrepancy (LLD), mechanical lateral distal femoral angle (mLDFA), medial proximal tibial angle (MPTA), were measured from the long radiographs, while lateral distal tibial angle (LDTA), and distal fibular station were measured off the AP and lateral tibia radiographs. The distal fibular station was defined as the distance of distal fibular physis relative to the talar plateau [13]. If the distal fibular physis was distal to the talar plateau, it was assigned a positive value, and if it was proximal to the talar plateau, it was assigned a negative value. Radiographs were also analyzed for latent changes, including refracture, migration of the male and female portions of the FD rods from their respective epiphyses, failure of the FD rod to telescope, and delayed axial deviation due to growth. This information was available on all limbs that underwent the procedure.

Statistical analysis was performed using GraphPad Prism (version 9.1.1). MPTA, LDFA and fibular station preoperative and most recent follow-up measurements were analyzed with Wilcoxon Rank Sum Test. LLD and mLDFA preoperative and most recent follow-up values were analyzed using a paired t-test. Findings were considered statistically significant if *p* < 0.05.

## 3. Results

All 39 CPTs achieved a complete union of the tibia and complete cross-union between the tibia and fibula following the index surgery. All but two patients also achieved a union of the congenital pseudarthrosis/osteotomy of the fibula. One of these persistent non-unions of the fibula was later united by surgical treatment at the time of planned rod exchange. No patient sustained a refracture of the tibia or fibula during the follow-up period (range 24–85 months).

Nineteen patients (20 limbs) also had postoperative long alignment radiographs 2 or more years after the index procedure available for analysis. The mean preoperative LLD was 21 mm (±26 mm), and the mean postoperative LLD was 14 mm (±17 mm). The LLD was unchanged (*p* = 0.2550). The mean pre-treatment mLDFA was 92° (±5°) and mean post-treatment mLDFA measured 88° (±2°), which was an improvement (*p* = 0.0021). The mean preoperative MPTA measured 93° (±4°), and the mean postoperative MPTA measured 91° (±3°), which did not demonstrate a meaningful change (*p* = 0.2859). Finally, the mean pre-treatment LDTA was 97° (±12°), and the mean post-treatment LDTA was 88° (±8°), which was an improvement both statistically and clinically (*p* = 0.0085) (Table 1).

The fibular station was analyzed in all 39 limbs. Fibular station was classified according to the Paley classification as a-or-b-types (2a-or-b, 4a-or-b). Preoperatively, there were 20 a-type fibulas (distal fibular physis at the station) and 19 b-type fibulas (distal fibular physis proximally migrated). The mean fibular station in the entire cohort was −3 mm (±4 mm) preoperatively and −2 mm (±3 mm) at the most recent follow-up, which was not an improvement (*p* = 0.661). The sub-analysis of the a-types demonstrated a mean preoperative fibular station of 0 mm (±2 mm) and a mean most recent follow-up fibular station of −2 mm (±3 mm), which migration of the fibula proximally (*p* = 0.0022). Analysis of the b-types showed a mean preoperative fibular station of −6 mm (±2 mm) and a mean most recent follow-up fibular station of −3 mm (±3 mm), which demonstrated an improved position of the fibula (*p* = 0.0038). When comparing most recent follow-up fibular station to normal (0 mm) there was statistically significant proximal migration for all types (*p* < 0.0001), a-types (*p* = 0.0159), and b-types (*p* = 0.0005).

Intraoperative transfusion of packed red blood cells was required in 24 of 36 patients. There were five postoperative complications. One patient developed skin necrosis medial to the incision over the subcutaneous border of the distal tibia. This required debridement and skin grafting. There were four complications related to the FD rod: one proximal end backed out into the knee joint requiring reinsertion; three procedures were needed to revise the distal locking wire due to prominence or backing out.

One patient required revision bone grafting and BMP2 insertion for a fibular delayed union, which was conducted at the time of a planned Fassier-Duval (FD) rod exchange. One patient who achieved a solid cross-union and still had a visible tibial osteotomy line six months later had percutaneous insertion of BMP2 at the time of the planned tibial plate and screw removal (all other patients tibial osteotomy line at the level of CPT disappeared by 12 weeks, with cross-union bridging radiographically present by six weeks after surgery). The osteotomy line disappeared after this minor procedure.

Fourteen patients underwent late elective surgical procedures to treat deformity or leg length discrepancy of the upper or lower tibia: ten insertions of hemiepiphysiodesis plates for guided growth; one acute correction of ankle valgus with osteotomy, bone grafting, and internal fixation combined with the planned FD rod exchange; two lengthenings to treat the leg length discrepancy, one with external fixation and one with an implantable lengthening nail; and one epiphysiodesis of the contralateral leg to treat the leg length difference.

**Figure 3:**
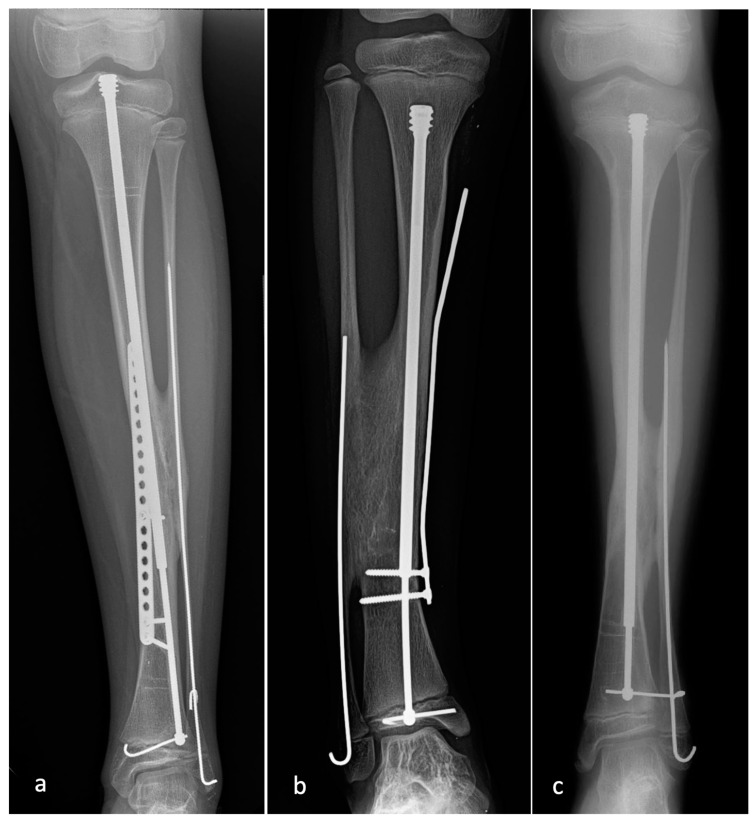
Radiographs demonstrating the various forms of FD rod migration: (**a**) the rod has pulled out of the distal epiphysis; (**b**) the rod has pulled out of the proximal epiphysis; (**c**) the rod has pulled out of both proximal and distal epiphyses. Note the reconstitution of the physis despite the breach by the FD rod.

Of the FD rods placed at the time of the index CPT surgery, 26/39 rods (66.7%) were noted to have pulled out of one or more epiphyses. Nine rods migrated across the proximal tibial physis, fifteen rods migrated across the distal tibial physis, and two rods migrated across both physes (Figure 3). Five of twenty-two rods (22.7%) placed at the time of the planned rod exchange also demonstrated migration. Two rods migrated across the distal physis, and three rods migrated across both physes. This did not lead to any additional surgery and was addressed at the time of the planned rod exchange in all cases. Despite rod migration across the physis, no growth arrest was seen in any patient in this study group.

## 4. Discussion

There have been many surgical techniques used to treat patients with CPT. In the past 30 years, the most common methods are: free vascularized fibular grafting, intramedullary (IM) nailing with bone grafting, Ilizarov external fixation, combined Ilizarov and bone grafting, and amputation [9,14,15,16,17,18].

Paley et al. [19] reported on 15 patients who had 16 tibiae with congenital pseudarthrosis. The mean patient age was 8 years, the rate of union was 94% in 15 patients with Ilizarov frames, refracture occurred in five tibiae (31%), and the mean follow-up duration was 4 years.

Boero et al. [20] reported on 21 patients with neurofibromatosis treated with Ilizarov frames. The mean patient age was 8.8 years. The primary union rate was achieved in 17 of 21 (81%) patients. Refracture occurred in four of the 17 patients (19%), and the minimum follow-up duration was 2 years.

The European Paediatric Orthopaedic Society (EPOS) multicenter study [15] of 340 patients with CPT reported a 75% healing rate achieved with Ilizarov external fixation and recommended the use of prophylactic IM rodding to prevent refracture.

In a series of 17 tibiae with CPT treated by Paley and Herzenberg, half of which were followed up to skeletal maturity, the mean patient age was 8 years, CPT union was obtained in 100% of the patients, and refracture occurred in 68% when the Ilizarov device without IM rodding was used [5]. When IM rodding was combined with external fixation, the refracture rate dropped to 29%.

Ohnishi et al. [21] reported 73 cases that were treated with different treatment protocols: 26 with Ilizarov fixation, 25 with vascularized fibular grafting, 7 with the combination of the previous two techniques, 6 with IM rodding combined with free bone grafting, 5 with plating and grafting, and the remaining 4 with different treatment protocols. The average patient age was 5 years. CPT union was achieved in all patients treated with Ilizarov fixation (four experienced refracture), 22 of 25 (88%) patients treated with free vascularized fibular grafting (one experienced refracture), and all patients treated with both fibular grafting and Ilizarov fixation.

IM rodding is an alternative treatment option to achieve and maintain the union, although the reported results are variable. Joseph and Mathew [22] reported 14 skeletally immature patients treated with IM rodding and double onlay autogenous bone grafting from the opposite tibia. The mean patient age was 4.5 years. The union rate was 86% and the mean follow-up was 3 years with a refracture rate of 21% (three of 14).

Johnston [23] reported on 23 patients treated with different techniques of IM rodding and grafting. The mean patient age was 2 years 4 months, the mean follow-up duration was 9 years, the primary union rate was 87%, and 13% had persistent nonunion and poor outcomes. The author identified two factors associated with the best outcomes were perfect limb alignment and the use of IM rods to achieve union, prevent refracture, and maintain alignment.

Kim and Weinstein [24] reported on 11 patients with 12 tibiae with congenital pseudarthrosis treated with IM rodding and free bone grafting. The mean patient age at the time of the index operation was 2.5 years. Four of the 11 patients healed after the primary index operation. Two of the four experienced refracture; one healed with a long lower limb cast, and the other healed after the index operation was repeated. The other seven did not heal after the index operation. Four of them achieved healing after undergoing multiple surgical procedures (one required free vascularized fibular grafting, and three required repeated IM rodding and grafting; one of the three had nonunion, one needed Syme amputation, and one had a failed Sofield procedure). Healing could not be achieved in the other three patients (two underwent below-knee amputation, and one had persistent nonunion at the latest follow-up visit). Kim concluded that IM rodding provides more predictable results in cases of late-onset pseudarthrosis [24].

Dobbs et al. [16] reported the long-term follow-up (mean follow-up duration, 14.2 years) of 21 patients with CPT (mean patient age, 5.1 years) treated with IM rodding and bone grafting. The primary union rate was 86% (18 patients), and three patients required additional bone grafting to achieve union. Twelve patients (57%) experienced refracture, and five (24%) required amputation.

Free vascularized fibular grafting had been described by several authors as a good option for achieving union in patients with CPT, although it is associated with many drawbacks, including nonunion, refracture, and recurrent nonunion at one site of the graft end [17,25,26,27]. Angular deformity of the affected tibia (valgus or anterior bowing) has been reported. The deformities usually are progressive and require further treatment [26,27,28]. Donor site morbidity, such as progressive ankle valgus with proximal migration of the distal fibula, is another problem associated with vascularized fibular grafting [26,27,28]. The tibiofibular synostosis can only delay but not prevent ankle valgus [26].

Weiland et al. [27] reported a 95% union rate in 19 patients after microvascular fibular grafting. Initial failure to achieve union occurred in 26% (five of 19 patients), and those patients required secondary procedures to achieve union (four healed and one underwent amputation).

Gilbert [25] reported the long-term follow-up of 29 patients who had CPT treated with microvascular fibular grafting, all of whom had reached skeletal maturity. The union rate was 94%, with a mean healing time of 6 months. The mean patient age at the time of the index operation was 5.5 years, the refracture rate was 14%, and the recurrence rate was 7%. Donor site morbidity occurred in 24%, tibial deformity (valgus and anterior bowing) occurred in 24%, progressive LLD occurred in 7%, and no amputation was recorded.

The EPOS study [29,30] reported a healing rate of 61% (19 of 31 patients) for vascularized fibular transfer. Seven of the 19 healed patients required additional procedures, such as grafting, plating, or IM rodding. The remaining 12 healed after the primary treatment and did not require additional surgery. Three patients (10%) required amputations, seven (23%) had not healed, and five (16%) experienced a fracture of the transferred fibula.

Toh et al. [31] reported seven cases of CPT treated with vascularized fibular graft, with a mean follow-up duration of 12.1 years. Casting or monolateral external fixation was used in the first cases; an Ilizarov fixator was used as a postoperative immobilization tool in one case. The author concluded that the best outcome could be achieved with combined vascularized fibular grafting and Ilizarov external fixation as a method of postoperative fixation.

El-Gamal et al. [18] reported three cases of CPT treated with vascularized fibular grafting combined with Ilizarov fixation to distract the fibular graft to correct LLD with a single operation. They called it ‘telescoping vascularized fibular graft.’ The mean patient age was 9 years, and the mean follow-up duration was 2 years. A union was achieved in all cases. One patient experienced refracture, and another patient experienced ankle valgus of the affected site.

Pharmacotherapeutic adjunctive treatment has also been employed as part of the treatment of CPT using BMP2, BMP7, and/or bisphosphonate therapy (ZA) [32,33,34]. Lee et al. [33] reported on 5 CPTs treated with BMP7 combined with allograft, IM rodding and external fixation. The mean age was 6 years, and the mean follow-up was 14 months. The use of recombinant human BMP7 was not enough to overcome the poor healing environment associated with CPT was the conclusion. Little et al. [16,32] used bisphosphonate (ZA) for patients with CPT to control the activity of osteoclasts to promote a union. The bisphosphonate was given after bone graft harvest so that it could not protect the bone graft bone from resorption.

Thabet et al. [35] conducted a retrospective study of 20 patients with CPT who were treated with periosteal grafting, bone grafting IM rodding of the tibia and fibula and circular external fixation. The mean age was 4.2 years. Eleven patients (55%) had NF1. Union was achieved in all patients (100%). The mean time spent in external fixation was 5.2 months (range, 3−12 months). Limb lengthening (mean 2.5 cm, range 0–7 cm) was simultaneously carried out in 12 patients. Refracture occurred in eight patients: one refracture in six and two refractures in two. Six of the eight patients with refracture had fibular pseudarthrosis. The mean time between the original surgery and refracture was 2.3 years (range, 1−5.8 years), and the mean time to second refracture was 4.7 years. All the refractures were united with additional surgery.

Amputation is either a primary or a last resort option in cases of CPT [9,14]. Its incidence varies from series to series. Foot condition, number of operations, and severity of LLD are the factors considered in making the decision for amputation [9].

The primary union rates range from 61% to 100% [8]. Refracture occurs 21 to 68% of the time, and up to 24% of patients were treated with Symes amputation. Failure of treatment is best represented by the rate of failure to achieve union combined with the refracture rate. Stated differently, the success rate is defined as the number of patients achieving union with the index procedure who did not refracture.

Paley [8] did a metanalysis of union, refracture, and success rates (union without refracture) of 25 published studies between 1990 and 2018. The studies were divided into four treatment groups: (1) rodding in ten studies (196 patients); (2) Ilizarov in six studies (115 patients); (3) Ilizarov plus rodding in five studies (152 patients); (4) free vascularized fibular graft in four studies (84 patients). The primary union was achieved in 61% of the rodding group, 93.5% of the Ilizarov group, 72% of the Ilizarov plus rodding group, and 66% of the vascularized fibula group. The refracture rate was 24% in the rodding group, 41% in the Ilizarov group, 17% in the Ilizarov plus rodding group, and 11% in the vascularized fibular group. Multiplying the union rate by one minus the refracture rate yielded the success probability of achieving union without refracture, which is the ultimate goal of surgery. The success probability was 40% for the rodding group, 57% for the Ilizarov group, 58% for the Ilizarov plus rodding group, and 58% for the vascularized fibular group. The average success probability of the combined 25 studies was 50.7%. A success rate of about 50% is not very reassuring to any parent consenting to CPT surgery treatment for their child, despite a thorough review of the literature going back 100 years.

This conclusion is further supported by two recent large studies [2,7]. Shah et al. [7] reported a long-term follow-up retrospective multicenter study of patients with CPT. Patients were treated with a variety of methods, including Williams rods, Ilizarov fixation, bone grafting, and free vascularized fibula. Union was achieved after the index procedure in 102/119 (86%). Amputation was used in 11/17 that failed primary union. Data regarding refracture was available on 94 of the primary union cases. Forty of these sustained a refracture (42.5%). The probability of union without refracture was 49.5%. The mean age at primary union was 5.6, and the mean age at refracture was 8 years. The refractured cases underwent 53 surgeries. At skeletal maturity, 82/119 were united (69%). A strong union was associated with no surgery on the fibula, the use of cortical bone graft, and either IM nailing or Ilizarov treatment. The combination of Ilizarov and IM nailing had a high rate of a weak union. The use of BMP was associated with a poorer outcome. Transfixation of the ankle was shown to improve the chance of obtaining union.

Kesireddy et al. [2] did a metanalysis reporting on 33 published studies of 401 cases of CPT. The mean age was 5.2 years, and NF1 was present in 262 (65%). The mean follow-up was 8 years. The mean rate of primary union was 75%, and the rate of refracture was 35%. The probability of union without refracture was 49%. The success probability of these two recent large studies was 49.5% and 49%, respectively, which was very similar to Paley’s metanalysis result of 50.7%. There appears to be a glass ceiling of success probability of approximately 50% with current methods of treatment.

Most recently, Laufer et al. [36] reported on 26 patients treated for CPT between 1997–2019. Six (Group A) were treated by resection of pseudarthrosis and bone transport with grafting of the docking site. Fifteen (Group B) were treated by resection of pseudarthrosis, acute shortening of the bone defect with grafting and lengthening of the tibia. Five (Group C) were treated by resection of the pseudarthrosis, acute shortening of the bone ends with grafting, and rodding with no lengthening. Group A had a 50% union rate with no refractures. Group B had an 80% union rate with a 33% refracture rate. Group C had a 60% union rate with no refractures. In total, 18/26 patients (69%) achieved union with a 22% refracture rate. These authors were the first publication to apply Paley’s success probability to a clinical study. They reported that the long-term success probability of union without refracture was 53.8%. They concluded that the surgical methods they used supported the assumption that a union without refracture could only be achieved in approximately 50% of cases. They also were the first other studies to use the Paley classification for the patients they treated: Paley type 1 in three patients (12%), type 2A in one patient (4%), type 3 in four patients (15%), type 4A in sixteen patients (62%), type 4B in one patient (4%), and type 4C in one patient (4%) [36].

Most studies report union and refracture rates, but few studies have examined functional outcomes after surgical treatment for CPT. Karol et al. published that patients had 68% reduced push-off strength if they had been treated with transarticular rodding across the ankle joint compared to 36% reduced strength when the rod did not cross the ankle joint [37]. Seo et al. reported that ankle function was well preserved after successful Ilizarov treatment of CPT [38], where no rod transfixed the ankle joint. Therefore, techniques that leave a rod across the ankle and subtalar joint are less desirable. This is the rationale in the Paley cross-union protocol for using an FD rod, which is fully contained in the tibia and does not span the ankle joint [12]. In contrast, the Choi et al. “4-in-1” cross-union method does recommend a rod across the ankle joint for better fixation [10].

Choi et al. [10] recommended the creation of a cross-union between the tibia and fibula for CPT only in cases where the fibula was broken but minimally proximally migrated. The two fibula bone ends were converged towards the tibia bone ends in what they called a “4-in-1 Osteosynthesis.” They used a cortico-cancellous sheet of the outer wall of the ilium combined with cancellous bone chips to achieve the cross-union. They did not recommend this method when the fibula was intact or when the fibula was significantly proximally migrated. They reported eight patients treated at a mean age of 6.3 years. All eight united with a cross-union to the fibula. There were no refractures at an average of 7.4 years follow-up (range 2.7–12.4 years). They compared to a group of 5 patients who had end-to-end repair of the tibia without cross-union. All 5 united and then refractured and required further treatment for the CPT. Choi et al. attributed the large cross-section of the bone at the level of the cross-union as the reason for no refractures. To quantitate this, they measured what they called the relative cross0sectional area (rCSA = area at the CPT site after union divided by area at the upper tibial physis). The rCSA was significantly lower in the non-cross-union group than in the cross-union group; 0.13 vs. 0.27 [10].

Paley [11] reported preliminary results dating back to 2007, using combined pharmacological and surgical management with cross-union. The protocol was similar to that used in this study but using a circular external fixator instead of a locking plate. All the rest of the protocol was the same. A larger study of the cross-union protocol with external fixation was reported separately [39,40]. Primary union and cross-union with the index procedure were achieved in 17/17 (100%). The total EF time was an average of 4 months (3–5). The mean radiographic union time was 4 months (1.5–6 mos). No refractures occurred in any of these patients. The calculated probability of union without refracture with this method was 100%, which is the same as in the Choi et al. series. Unpublished further follow-up of these 17 tibias shows no deterioration or refracture with up to 14 years (mean 7 years, range 6–14 years) follow-up. Although no gait analysis was conducted, at the last clinical follow-up, hip, knee, ankle, and gait functions were reported to be normal in all children. The rCSA in the Paley study was a mean of 0.46 ± 0.14 [11]. This rCSA is much higher than 0.27 reported by Choi et al. [10]. This is not surprising since the fibula and tibia in the Paley protocol are not converged as in the ‘4 in 1’ Choi et al. technique. To evaluate whether decancellousization of the ilium has any effect on hip development, the center-edge angle (CEA) and acetabular index (AI) were measured on the harvested versus unharvested side. There were no significant differences in CEA and AI between sides. This evaluation was not repeated in the current study, although no hip dysplasia has been noted.

As with the external fixation group, the all-internal fixation patients reported on here successfully obtained union of all tibias and demonstrated no refractures during a minimum 2-year and up to 7-year follow-up. The success probability of union without refracture of the previously reported external fixation cross-union protocol group and the current all internal fixation cross-union protocol group was 100%. This is in stark contrast to Shah et al. [7], Kesireddy et al. [2], and Paley [8], documenting that the probability of union without refracture was 49.5%, 49%, and 50.7%, respectively.

**Figure 4:**
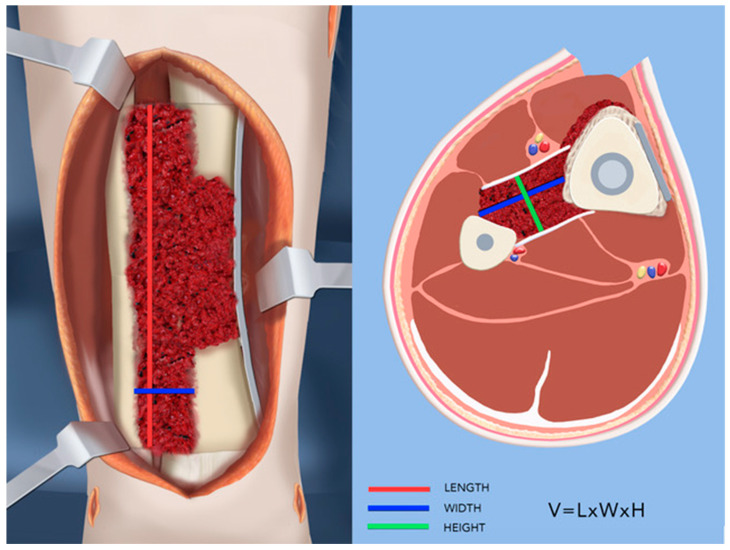
Illustration of the calculation of THE volume of bone graft needed: Volume (V) = the length of the planned cross-union (X) × the width between the two bones (Y) × the height of the space between the bones (Z).

How do we explain the increase in union rate to 100%? Other less successful methods, such as the Ilizarov technique, IM rodding, and vascularized fibular graft, all involve resection of tibia and treatment with bone grafting, bone transport or vascularized fibular replacement (referred to heretofore as ‘other methods’). The Paley cross-union protocol recommends against resection of the tibia since the bone of the tibia, although diseased, is viable. As previously noted, the bone of the tibia is damaged secondary to the fibrous hamartoma, which replaces the periosteum [41,42]. The bone of the tibia is not the primary cause of CPT. Therefore, resecting it does not cure the disease. In the cross-union protocol, the only bone resected is conducted so as to allow the bone ends to come together end to end when acutely correcting the angular deformity. Other methods resect the hamartoma of the tibia locally at the CPT site. The cross-union approach resects the hamartoma off the tibia and the fibula over a longer distance so that a lengthy cross-union can be created. If the fibular or tibial periosteum or hamartoma is left in place, the cross-union will not bridge to the fibula and/or tibia due to the interposing soft tissue. When the tibial and fibular pseudarthroses are at different levels, the hamartoma and periosteum need to be denuded from a few centimeters distal to the more distal pseudarthrosis to a few centimeters proximal to the more proximal pseudarthrosis to ensure there is no soft tissue interposition between the tibia and fibula that would prevent successful cross-union. Other methods use synthetic bone substitutes, allograft, or autogenous cancellous or cortical bone only around the tibial pseudarthrosis site. The cross-union protocol uses only autogenous cancellous bone. Allograft bone and bone graft substitutes are osteoconductive and/or osteoinductive. Autogenous bone is both conductive, inductive and productive due to the presence of living bone progenitor cells. Cancellous bone is considered superior to the cortical bone for osteogenesis [43]. In the cross-union protocol, the goal of grafting is to fill the space between the tibia and fibula opposing surfaces without specifically grafting around the pseudarthrosis sites of each bone. The volume of bone required to achieve this can be calculated simply from AP, lateral and mortis view radiographs by measuring the length of the planned cross-union (X) from the AP view, the width between the two bones (Y) from a mortis view, and the height of the space between the bones (Z) from a lateral view. Although a more accurate way might involve obtaining a CT scan or MRI, the added radiation or need to sedate the child are not indicated for this approximate calculation, which is used to guide the bone graft harvest. The volume (V) of bone graft needed is V = X × Y × Z (Figure 4) (e.g., in an 18-month-old child where the length = 6 cm, width = 1.5 cm, and height = 1.5 cm, the volume of graft required is 6 × 1.5 × 1.5 = 13.5 cc).

**Figure 5:**
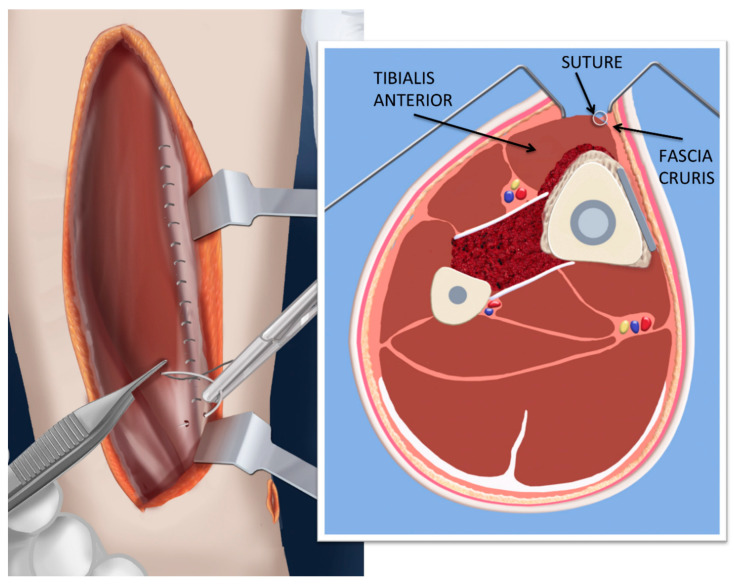
Illustration of the cross-sectional appearance of the cross-union. Note the BMP is the outermost layer with the bone graft contained between in the interosseous space. This allows the BMP to maintain contact with the vascularized soft tissues. The anterior compartment musculature is closed overtop.

This is a large volume of autogenous cancellous bone to procure, especially in a very young child. For this reason, other methods replace or augment autogenous bone with allograft or synthetic substitutes. This may contribute to the high failure rates of other methods. The author prefers to use all autogenous cancellous bone for the reasons previously discussed. The cross-union protocol uses a technique previously described by the senior author, called decancellousization of the ilium [8]. This method hollows out the entire iliac bone on one side of the pelvis and gives a much larger yield of cancellous autograft while preserving the cortical structure of the ilium. Typically, even as early as 12 months of age, between 15–20 cc of bone graft can be harvested by decancellousization of the ilium. The Paley cross-union protocol also prevents bone graft resorption, a problem that has plagued other methods, by giving Zoledronic acid 2 weeks prior to surgery to ensure that the bisphosphonate penetrates the bone graft before it is harvested. The cross-union protocol also places the BMP2 in a strategic location. Rather than placing it around the CPT site, the BMP2 is placed between the soft tissues and the bone graft. BMP2 induces bone by recruiting mesenchymal stem cells from vascularized soft tissues. The extensive hamartoma and interosseous membrane resection allow the BMP2 to sit directly on the posterior and anterior muscles and to recruit cells from the muscles to the bone graft. The cross-union protocol also addresses the lower level of BMP production in NF1 bone by having the BMP2 in contact with the bone graft, where it can upregulate stem cells in the autogenous cancellous bone to make new bone (Figure 5) [32].

**Figure 6:**
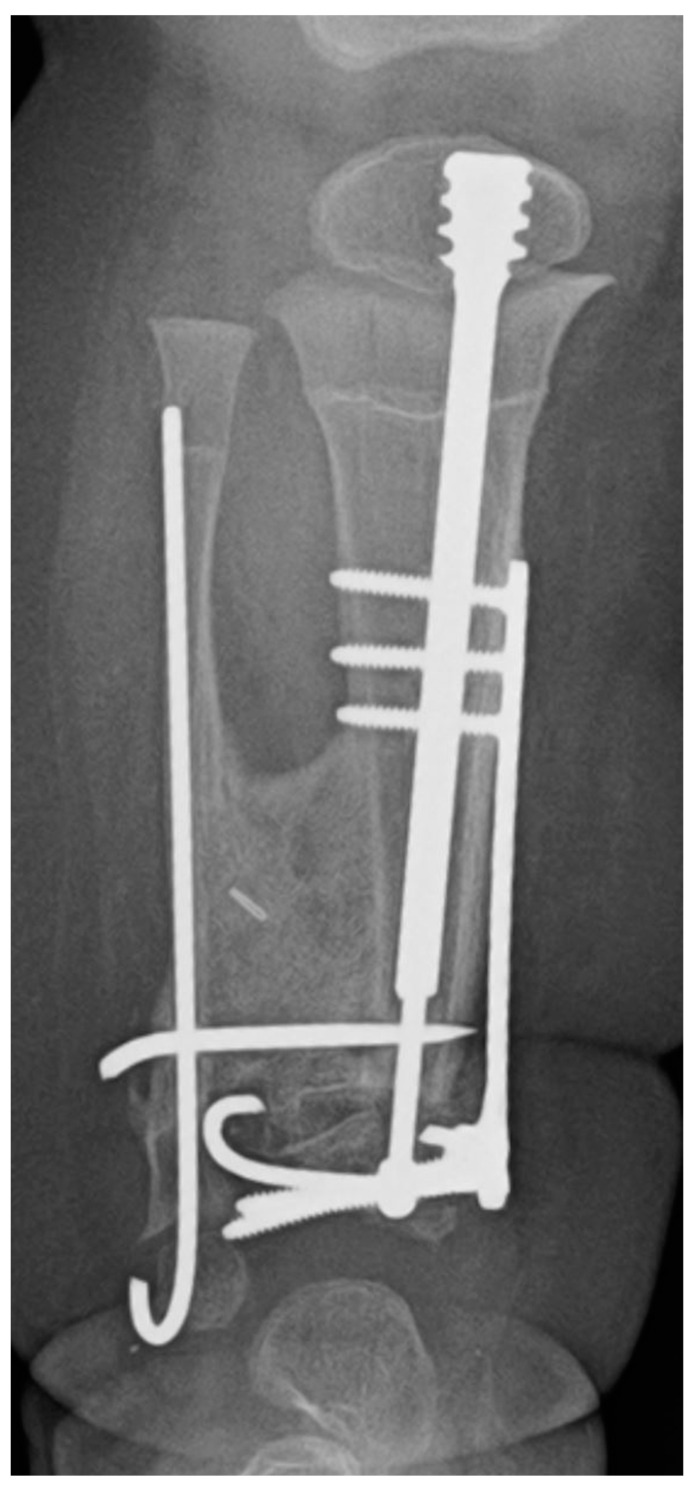
AP radiograph showing an example of a very distal pseudarthrosis site. This required fixation into the distal tibial epiphysis as there was not enough distal tibial metaphysis for screw placement. The cross-union is fully healed, and these screws can now be removed to allow the physis to resume growth.

The cross-union protocol did work without BMP2 in one case in this study in which BMP2 was contraindicated due to a glioma. The senior author has also succeeded with the cross-union protocol for CPT when operating outside the USA in places where BMP2 is not available. The advantage of using BMP2 may be to accelerate the osteogenic response. Most of the cases showed cross-union bridging as early as six weeks after surgery. Finally, the fixation used in the cross-union protocol is greatly superior to other methods that only use a rod in the tibia. The combination of rodding of the tibia and fibula provides additional stability to both of these bones. Furthermore, the addition of a plate on the tibia with the nail offers compression and rotational stability of the tibia. Therefore, the stability offered by these implants far exceeds that of other methods. Due to this stability, there is no reason to pass pins or rods through the ankle, which can lead to permanent damage and stiffness of the ankle joint [37,38]. Even in very short tibial segments, the plate fixation can extend with screws into the distal tibial epiphysis using a T-plate to temporarily span the distal tibial physis (Figure 6). The epiphyseal screws are removed 6 weeks later. The only failure we experienced in achieving union was at the fibula in two cases. This was also previously reported by Paley in 8% of cases [8]. This complication is likely related to the lack of preparing and filling of the interosseous space in very distal fibular pseudarthrosis cases. To prevent persistent fibular nonunion, the periosteum between the two bones should be removed on the opposing surfaces of both bones down to the level just proximal to the distal tibial physis. It is important to pack autogenous grafts into this interspace.

**Figure 7:**
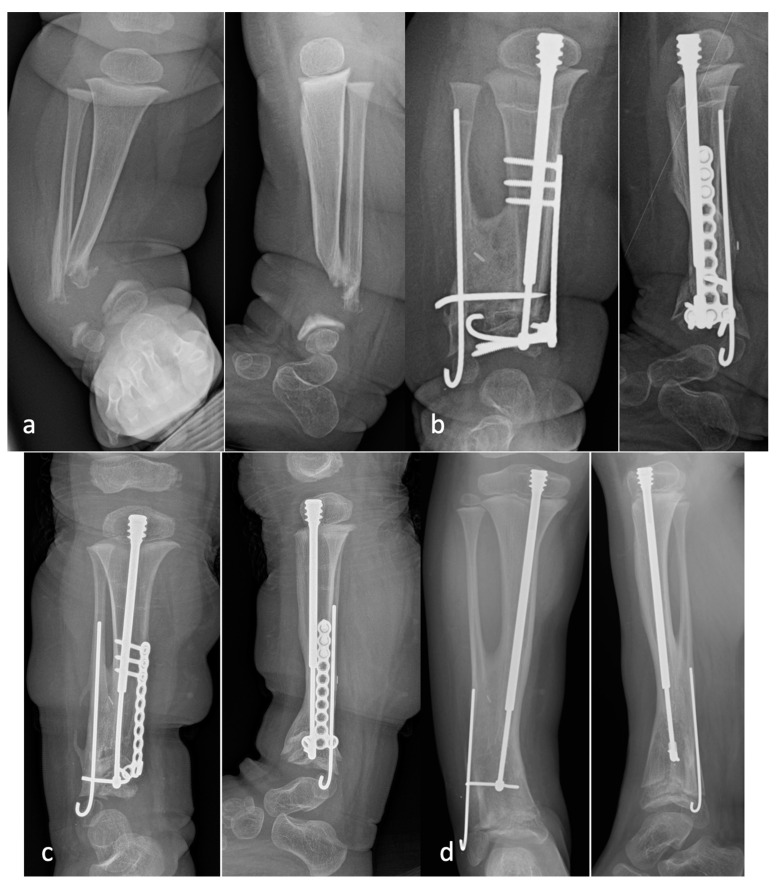
Radiographs of a patient who underwent a cross-union with internal fixation: (**a**) preoperative AP and lateral radiographs demonstrating a large bone defect and small distal metaphyseal segments of the tibia and fibula (Type 4C); (**b**) AP and lateral radiographs taken 12 weeks after surgery show a well-healed cross-union with bridging between the tibia and fibula, as well as filling of the bony defects; (**c**) AP and lateral radiographs taken 1 year after surgery with growth of the bone at both ends and telescoping of the FD rod to the level of the cross-union; (**d**) AP and lateral radiographs taken at 2.5 years after surgery, after a one-rod exchange, with a solid cross-union and no refracture.

How do we explain the 0% refracture rate of the tibia and fibula? This is most likely related to the biomechanics of the cross-union. The bony union of the tibia and fibula increases the cross-sectional diameter of the bone in the area of pseudarthrosis. The torsional rigidity of a bone is proportional to the radius to the 4th power, and the bending strength of a hollow cylinder is proportional to the radius to the 3rd power [44]. Both of these numbers, and therefore, the strength of the bone to resist torsion and bending, are dramatically increased as the diameter of the bone increases. The cross-union achieves this increase in diameter by linking the two bones together, with additional stability provided by the use of intramedullary rodding. With a rod filling the canal of both the tibia and fibula, the bending strength of the cross-union can now be calculated as a solid cylinder, which is proportional to the radius to the 4th power. The exponential increase in the leg’s structural stability leads directly to a decreased risk of fracture, as demonstrated by the complete lack of refractures. These biomechanics also explain our rationale for using a growing rod to ensure that all areas of the bone have a prophylactic rod in situ despite growth. This is the same rationale as using the FD rod in osteogenesis imperfecta [45]. The tibia in CPT should be considered potentially fragile without the rod spanning its entire length. This also explains the rationale for changing the FD rod to a longer and larger diameter rod before the female larger diameter part of the rod migrates past the original CPT level, leaving only the smaller diameter male rod to protect the bone. Refracture is also mitigated by giving a second dose of Zoledronic acid 3 months after the cross-union surgery so that the newly formed bone is also protected from osteoclastic catabolism (Figure 7).

During this study period, planned FD rod exchange occurred once in 22 tibias and twice in one tibia. The timing of rod exchange ranged from 7 to 35 months following the index surgery. We compared the interval to rod exchange for children treated between ages 1–2 years old vs children between ages 2–4 years old vs children over the age of 4. The average interval to rod exchange in the 1–-year-old group (*n* = 8) was 19 months vs. 29 months in the 2–4-year-old group (*n* = 10) vs 20 months in the over 4-year-old group (*n* = 4). This difference was significantly different *p*= 0.005 between the 1–2-year-olds and the 2–4-year-olds. We could not compare to the over 4-year-old group due to the small sample size. There was no significant correlation found between age at index procedure and time to rod exchange when looking at the entire group and the subgroups. Most other elective procedures such as hemiepiphysiodesis were planned to coincide with the timing of rod exchange to avoid performing additional anesthetics.

Although a telescopic rod offers many advantages in the growing child, as discussed above, there are also some potential problems with this implant. The most common problem is pulling out of the epiphysis with distal migration of the proximal end and proximal migration of the distal end. In this study, out of a total of 39 rods placed at the index procedure, nine rods migrated distally at the proximal end, one rod migrated proximally at the proximal end, and fifteen rods migrated proximally at the distal end. Two tibias had the rod migrate at both ends. Five rods redeveloped migration after the initial rod exchange. Two of the rods pulled out at the distal end, and three rods pulled out at both ends.

The reason for migration is clear, while the cause may not be evident. The rod will migrate anytime the force on one end of the rod is less than the force for the male and female parts of the rod to separate. The rod is held proximally by screw threads in the proximal tibial epiphysis. It is held distally by a locking pin in the distal tibial epiphysis. The interlocking and friction forces of the screw threads in the proximal epiphysis or the cross-locking wire in the distal epiphysis should be greater than the force of two, low-friction, polished stainless-steel surfaces sliding apart. Therefore, as the epiphyses move away from each other by physeal growth, the rod should telescope freely with little resistance. There are two factors that can change this balance: (1) stiction and (2) external impingement on the rod. In the first instance, a bending force will cause the rod to be loaded in bending. Such bending leads to stiction, which is defined as the sum of static friction that a body is needed to overcome to initiate motion [46]. If the stiction at the telescopic portion of the rod is greater than the stiction of the proximal threads or distal locking wire, then the movement will occur at the path of least resistance, which becomes migration of the proximal or distal ends of the rod from the epiphysis. Such bending force may arise from either the growth plates growing asymmetrically or the weak diaphyseal bone trying to bend and being resisted by the rod. This is seen in other methods of treatment for CPT and probably accounts for the migration of the Williams rod out of the foot and ankle. It is also evidenced by the transosseous migration of the rod out of the diaphysis of the bone as the bone bends or grows away from it, presumably through Wolff’s law of remodeling [47]. Paley noted that the rod tended to pull out of the epiphysis more in patients who developed a valgus deformity of the upper tibia [8]. We could not corroborate that in this study. The same could occur when the distal tibia grows into varus or valgus. This should be referred to as secondary stiction since it is not present at the time of rod insertion. The second cause, external impingement on the nail, is related to impingement on the nail from screws of the plate. If any of the screws touch the rod, they can create friction to sliding and impingement. This makes it difficult for the rod to slide within the bone, thus creating drag on the rod. This mechanism should most frequently affect the proximal epiphyseal migration. Most of our cases pulled out of the distal epiphysis and not the proximal epiphysis. On the other hand, if the screws are indenting the rod enough to incarcerate the male rod within the female, we should expect to see the rod pull out of both ends. It is very difficult to determine screw impingement radiographically. It can be diagnosed after the fact at the time of rod exchange. If there are scratch or indentation marks on the rod at one or more levels, then there was screw impingement from the time of inserting the plate. Removing the screws of the plate early as recommended by Paley for the dynamization of the rod should alleviate any possible impingement and remedy the problem if it is not too late [8].

The FD rod was recently modified by the manufacturer (Pega Medical, Montreal, QC, Canada) to help address these issues. Previously, the distal locking hole in the male component was 1.6 mm in diameter for all rod sizes, allowing for locking with a 1.5 mm wire. The male component has been modified to allow for larger diameter locking pins proportional to increasing diameters of the male rod. These larger diameter locking pins are stiffer and require more force to bend and pull through the distal tibial physis. The proximal end has also been modified by adding a locking hole of a similar diameter. This, too, can be locked with the same size pin or peg. To avoid blocking this hole, the male component should be cut about 1 cm shorter than the total length of the tibia. We have only recently started to use this newer FD rod and therefore do not yet know if this will prevent the high rate of rod migration seen.

**Figure 8:**
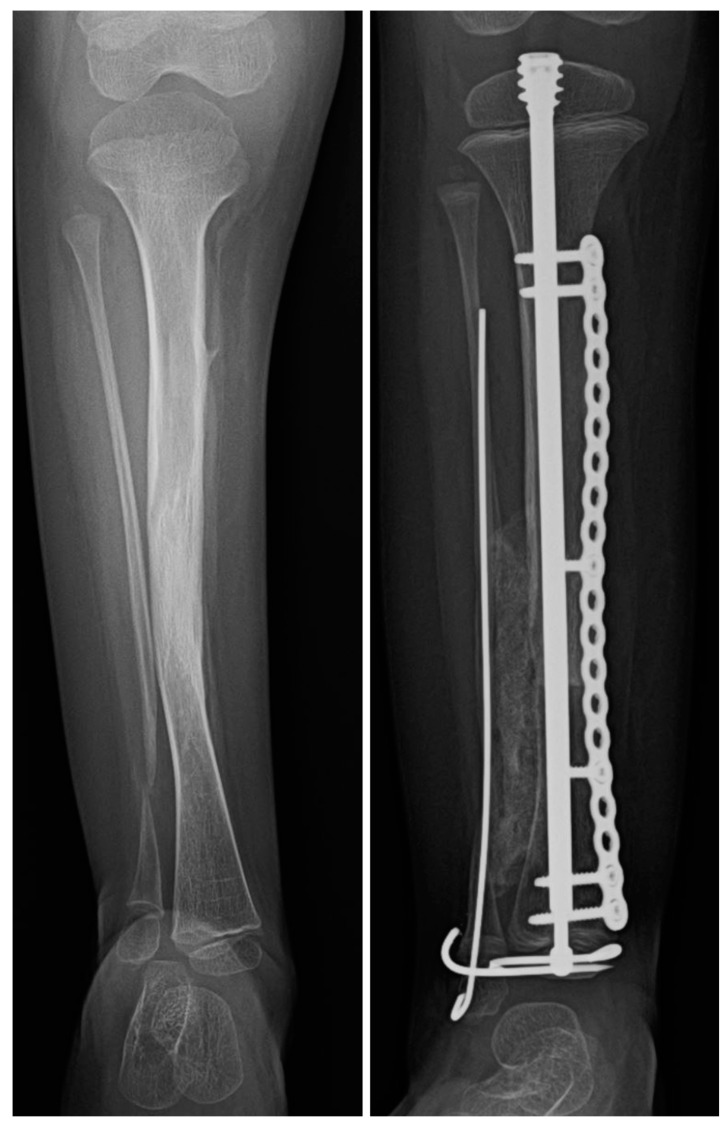
The image on the (**left**) demonstrates the proximal fibular migration that can occur as a secondary change in CPT (type 2B). The image on the (**right**) shows the restoration of the fibular station after the cross-union procedure with fibular distalization. The fibula is brought down and stabilized with a wire fixing it to the tibia in the corrected position.

Proximal migration of the distal fibula is a common problem seen in patients with CPT. Migration of the fibula is related to fibular fracture and the development of valgus of the ankle plafond [48,49,50]. This subsequently leads to deformity, instability, and degeneration of the ankle joint. Choi classified the fibular migration separately [10]. Paley incorporated fibular migration into his CPT classification, designated with a-or-b modifier after the type number [11]. Paley was also the first to recommend treating the fibular migration surgically at the time of the CPT repair [11] (Figure 8). Interestingly, the b-type fibulas that were distalized at the time of cross-union maintained their corrected fibular station 83% of the time. This demonstrates that moving the fibula distally in 2b and 4b cases is effective. On the contrary, in a-type CPT, 35% of fibulas were proximally migrated at final follow-up with an average proximal change of 3.7 mm. Studies of post-traumatic cross-union of the tibia and fibula have demonstrated proximal migration of the fibula due to the differential growth rate of the tibia and fibula [51,52]. The distal tibial physis grows faster than the distal fibular physis. Accordingly, one would expect that all type-a fibulas would migrate proximally. This did occur in 7/20 of cases. We continue to monitor this to see if this will become a problem secondary to creating an intentional cross-union.

The primary problems in CPT are (1) anterolateral bowing, (2) non-healing fracture (pseudarthrosis), and (3) proximal migration of the fibula. As a consequence of these three primary conditions, a myriad of secondary deformities develop. Most of the secondary conditions are due to the effect of the primary condition on the surrounding soft tissues and joints and the secondary effects on the growth and development of the lower limb. The anterolateral bowing relaxes the posterior muscles leading to decreased tension on the Achilles tendon. This leads to atrophy and thinning of the calf muscles and eventually to a calcaneo-cavus deformity of the foot with a pistol grip heel. The anterior bow of the tibia causes the foot to assume a dorsiflexed position. The anterior soft tissues fail to elongate, and the anterior capsule is never stretched into equinus. This leads to a dorsiflexion contracture of the ankle (calcaneus deformity of the foot). The proximal migration of the fibula causes the talus to follow the fibula. This leads to lateral subluxation of the ankle joint and valgus instability of the ankle. The distal tibial plafond becomes wedge-shaped relative to the distal tibial physis. The wedging of the distal tibial epiphysis produces a valgus plafond orientation. The ankle valgus serves to compensate for the lateral bowing, which in effect is a varus distal tibial deformity. Similarly, the proximal tibial physis grows into recurvatum and valgus to compensate for the procurvatum-varus diaphyseal deformity. A recent publication by Deng et al. demonstrated that persistent pseudarthosis of the fibula and shortening of the fibula, even in the setting of tibial union, resulted in a statistically significant ankle and knee valgus compared to patients who had an intact fibula that maintained station [50]. The lack of loading on the tibia and the altered muscle forces, as well as the proximity of the pseudarthrosis to the distal tibial physis, lead to the slowing of the growth of the distal tibial and fibular physes and leg length discrepancy. In response to the altered forces in the lower leg, the proximal femur responds by growing into coxa valga [53]. The coxa valga may explain the overgrowth of the femur despite the undergrowth of the tibia. CPT is one of the few conditions with developmental leg length discrepancy (LLD) that compensates for overgrowth in the femur. In some cases, the coxa valga can be so extreme that it leads to hip dysplasia. The LLD, foot, ankle, knee, femur and hip deformities are all secondary problems associated with CPT.

Fixing the primary problems in patients with CPT is the primary goal of treatment. The secondary problems are usually only addressed if the primary nonunion and angular deformity of the tibia can be successfully treated. Therefore, the primary objectives of treatment for patients with CPT are: (1) Straighten the anterolateral bowing at the CPT site; (2) *obtain* and *maintain* the union of the tibia at the CPT site; (3) Obtain union of the fibula to prevent or treat proximal fibular migration. The secondary objectives are to prevent or treat the secondary deformities of the ankle and foot and the leg length discrepancy.

**Figure 9:**
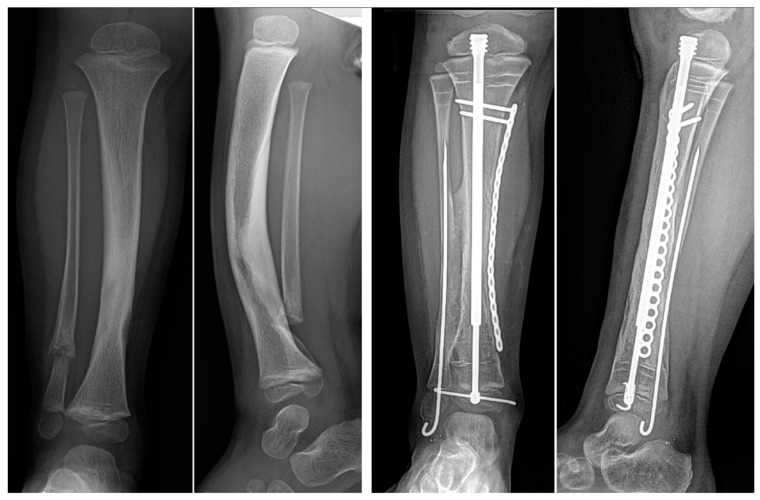
AP and lateral radiographs of a patient with Paley type 2A CPT. The preoperative images on the left show an intact tibia with fibular pseudarthrosis. A tibial osteotomy was made at the apex of the deformity, straightened, and fixed with the standard cross-union protocol. The postoperative images on the right show a well-healed osteotomy and cross-union with normal tibial alignment.

The treatment of patients with pre-CPT (Paley type 1 and 2) by the cross-union protocol is controversial. The conventional treatment for pre-CPT cases has been to brace them as long as possible to prevent fracture. Non-operative treatment likely does not prevent the development of the secondary changes of atrophy, leg length discrepancy, fibular migration, ankle valgus and calcaneus, cavus foot, coxa valga, etc. Since straightening the tibia in pre-CPT puts the tibia at risk of developing a pseudarthrosis of the osteotomy site, the treatment of pre-CPT has been non-operative until the tibia fractures despite the development of secondary changes. Laufer et al. treated 4/26 patients with pre-CPT (Paley 1 in three and Paley 2a in 1) [36]. All four obtained union, and one refractured. With the cross-union protocol results showing a 100% success probability in actual CPT cases, it was a reasonable calculated risk to do the same treatment for cases where the tibia is not yet broken in the belief that this would mitigate against the development of secondary changes. For this reason, the senior author chose to treat five type 1 and seven type 2 cases where the tibia was intact. These patients had an osteotomy of the tibia at the diaphyseal apex of angulation and the rest of the treatment as per the cross-union protocol (Figure 9). All united, and none refractured. Therefore, cross-union surgery is a reasonable option in patients with Paley type 1 and 2 CPT and should prevent secondary changes to the foot, tibia, and femur from developing in this group of patients.

Recently guided growth was proposed as a treatment method in young patients with intact anterolateral bowing to prevent the development of fracture and pseudarthrosis and to correct deformity early [54]. The preliminary results appear promising, with all ten patients in the series avoiding any tibial fracture and obtaining a significant improvement in the bony alignment. Further follow-up is needed to determine if fracture and secondary changes of leg length difference, ankle, and foot deformity will be prevented with this technique. The senior author treated the patients with intact pre-CPT in this series before this new method was published. The guided growth method is certainly appealing since it is minimally invasive compared with performing a cross-union. It seems there is little to lose in first trying guided growth for these pre-CPT cases as it does not seem to burn any bridges. The cross-union would remain as a back-up procedure in case of failure.

There was a very high transfusion rate related to blood loss from the decancellousization of the ilium in this study: 24/36 (67%). The CPT surgery is performed using a tourniquet on the leg for up to 2 h and therefore did not contribute much to the blood loss. None of the patients had any adverse outcomes related to the transfusion. Recognition of this high transfusion rate has led us to modify our protocol to include tranexamic acid (TXA) prior to the start of the surgery [55], the use of autogenous blood salvage (Cell Saver, Haemonetics Corporation, Braintree, MA, USA), infusion of Venofer (intravenous iron), and a lower threshold for transfusion (<Hgb = 6). To see if this change in protocol reduced our transfusions, we reviewed the records regarding transfusion in the 18 additional patients who underwent cross-union protocol surgery for CPT with all-internal fixation, subsequent to the study cohort. All 18 patients were excluded from this study due to less than 2 years follow-up. As with the study cohort, all 18 achieved CPT union and cross-union. Only three of these 18 patients (17%) required transfusion. Seventeen (94%) of these patients received TXA, and 16 (89%) received an immediate postoperative Venofer infusion. The decrease in transfusion rate from 67% to 17% clearly demonstrates that the decancellousization does not have to lead to such a high transfusion rate. Modifications to the blood management protocol can be successful in reducing the need for blood transfusion. Further study of this more recent cohort is needed to look at this issue more critically.

Cross-union has been reported by Choi et al., as noted previously, using what is called the 4-in-1 Osteosynthesis [10]. They also demonstrated 100% union with no refracture (8/8 with a 7.4-year follow-up). Two other recent reports of cross-union treatment for CPT using modifications of the Paley and Choi methods show similar results: Vaidya et al. [56] reported 10/10 patients healed with no refracture (100%), and Liu et al. [57] reported on 17/17 patients healed with no refracture (100%) at 4-year follow-up. In addition an as-of-yet unpublished study (personal communication) by Dr. Bo Ning from Fudan University, Shanghai, China, reports on 18 patients with CPT treated by Paley cross-union protocol using all internal fixation, with 18/18 achieving CPT union with 0% refracture during a mean follow-up of 4.3 years (range 1.5–6.3 years) [58]. Longer-term follow-up and more corroboration of these results are expected.

This study is not without limitations. This is primarily a radiographic review, which does not provide any information about the long-term function or patient reported outcomes. Due to the previous difficultly in achieving and maintaining union with CPT, there are few such functional outcome studies [37,38,59]. Therefore, the standard has been to primarily report union and refracture rates as the measure of success of a surgical method in the treatment of CPT. We have followed that standard. We have demonstrated that our success rate is much higher than that reported for other methods. We have limited the variability since all of the surgeries were performed by one surgeon at one institution using the same protocol. It remains to be seen whether these results will hold when single-center or multicenter studies using this protocol publish their results.

## 5. Conclusions

Cross-union for patients with CPT represents a paradigm shift in the management of this sinister pediatric ailment. The surgical technique is demanding but reproducible, and careful attention to blood loss management is needed. The dramatic change in success rate from 50% literature wide, to 100% with cross-union, cannot be ignored. With such a large improvement, consideration should be given to eliminating less successful, older methods and making cross-union the new standard for treatment of CPT while remaining open-minded to improvements that may come along in the future.

## Figures and Tables

**Table 1:** Postoperative joint orientation angles and leg length discrepancy at followup.

	Pre-Operative	Final	*p* Value
mLDFA	92° (±5°)	88° (±2°)	0.0021
MPTA	93° (±4°)	91° (±3°)	0.2859
LDTA	97° (±12°)	88 (±8°)	0.0085
LLD	21 mm (±26 mm)	14 mm (±17 mm)	0.2550
